# Monochorionic Twins and the Early Mother-Infant Relationship: An Exploratory Observational Study of Mother-Infant Interaction in the Post-Partum Period

**DOI:** 10.3390/ijerph19052821

**Published:** 2022-02-28

**Authors:** Chiara Ionio, Eleonora Mascheroni, Gianluca Lista, Caterina Colombo, Giulia Ciuffo, Marta Landoni, Irene Daniele, Merideth Gattis

**Affiliations:** 1 CRIdee Unità di Ricerca sul Trauma, Psychology Department, Università Cattolica del Sacro Cuore, 20123 Milan, Italy; 2 0–3 Center for the at-Risk Infant, IRCCS Eugenio Medea, 23842 Lecco, Italy; eleonora.mascheroni@lanostrafamiglia.it; 3 Neonatologia Patologia e Terapia Intensiva Neonatale, Ospedale dei Bambini “Vittore Buzzi”, ASST Fatebenefratelli Sacco, 20154 Milan, Italy; gianluca.lista@asst-fbf-sacco.it (G.L.); caterina.colombo@asst-fbf-sacco.it (C.C.); irene.daniele@asst-fbf-sacco.it (I.D.); 4 CRIdee, Psychology Department, Università Cattolica del Sacro Cuore, 20123 Milan, Italy; giulia.ciuffo@unicatt.it (G.C.); marta.landoni@unicatt.it (M.L.); 5 School of Psychology, Cardiff University, Cardiff CF10 3AT, UK; gattism@cardiff.ac.uk

**Keywords:** preterm birth, monochorionic twins, parenting, mother-infant relationship

## Abstract

The extraordinary increase in twin rates and specifically monochorionic twin pregnancies represents a major public health issue due to the associated increased risks for the mother, the child and their relationship. The aim of the present study was to examine the quality of mothers’ behaviour during mother–infant interaction in the early postpartum period by comparing mothers of twins and mothers of singletons during face-to-face interaction with their infants. Demographic and clinical information was collected by trained research psychologists from the mothers’ and the childrens’ clinical records and from interviews with the mothers. At three months (corrected for prematurity), the interactions of the dyads (11 mother-twin infant dyads and 11 mother-singleton dyads) were filmed at participants’ homes in accordance with the procedure of the Global Rating Scales. Maternal behaviour during interactions was assessed and rated by two trained research psychologists. With regard to the mothers’ interaction with each twin, no differences were found between mothers’ scores in every GRS subscale, indicating that mothers did not interact differently with their twins. Comparisons between mothers of MC twins and mothers of singletons showed that the quality of maternal sensitive behaviour during the interactions were lower in mothers of twins (0.35) Mothers of twins were also more distant during interactions with their babies and more likely to experience depression symptoms than mothers of singletons (0.05). Future research should examine mother-infant relationships following twin birth with larger samples. Such research will be especially useful in evaluating the potential benefits of interventions to promote positive mother-infant interactions.

## 1. Introduction

Twin pregnancies account for approximately 3% of all live births [1]. This extraordinary increase in twin rates is an important public health issue because twin pregnancies are generally associated with increased risk to the infants and the mothers [2]. In particular, monochorionic twin pregnancies (MC) account for 20–25% of all twin pregnancies, and their incidence is steadily increasing [3]. Their morbidity and mortality rates are higher than dichorionic (DC) pregnancies due to specific complications [4,5]. Monochorionicity is associated with an increased incidence of preterm birth [6], low birth weight [7] and a longer stay in the neonatal intensive care unit (NICU) [8]. Moreover, the prevalence of congenital anomalies is almost twice as high when comparing MC twins with DC twins, although in both cases, only one foetus is affected in 90% of the cases [9].

Because MC twin pregnancies are associated with multiple risk factors, pregnant women reflect on their pregnancy and classify it as a “high-risk pregnancy”, which affects how the woman perceives and lives the pregnancy and future bonding with the child [4]. Studies of prenatal bonding in twin pregnancies have found that zygosity (i.e., the degree of genetic similarity in twins) is an important factor in the relationship between the mother and her twins [10,11]. All MC twins are monozygotic twins (MZ). From the genetic point of view, dizygotic twins (DZ) are fraternal twins, while MZ twins are identical twins. Specifically, it has been observed that women expecting monozygotic twins tend to imagine the twins as a pair with similar characteristics whereas, women expecting dizygotic twins imagined the twins as individuals with different characteristics [12].

As far as we know, there are no studies that have investigated the influence of zygosity on the early interaction between mother and twins. However, based on the above considerations, it can be hypothesised that MC twins, mothers tend to focus the mother–child relationship on the twin pair as a whole, without focusing on the characteristics and needs of each individual child.

During a typical pregnancy, mothers use their representations of the unborn child, of themselves as future mothers and of their relationship with their own parent figures to prepare for motherhood [13]. In these complicated pregnancies, the expectant mother may suffer shock, and become unable to think or project for herself; maternal fantasies may be blocked. Bonding during pregnancy is a crucial aspect of the parent–child relationship. It is the pregnant woman’s investment in her unborn child. It evaluates the mother’s ability to adopt behaviours associated with foetal belonging and to interact with her unborn child [14]. The novelty of this concept is that it can be used to describe a particular aspect of the mother–foetus relationship based on conscious representations. These include cognitive representations of the mother’s interactions with her unborn child, scenarios showing that the mother is able to assign physical and emotional characteristics to her foetus (recognising that the foetus is different from the mother) and dreams and expectations for the unborn child. Certain maternal behaviours also reflect this investment, such as the changes women make to ensure the foetus’s health.

MZ birth is a problematic event that disrupts the establishment of parental mental representations and is a potential risk factor not only for child development but also for the construction of parental functioning and a positive mother–child relationship [15,16,17,18,19,20,21,22]. In addition, MZ birth is generally associated with high levels of maternal stress, anxiety and depression, as well as feelings of inadequacy [23,24], ([25] pp. 61–68), [26,27,28]. As reported in the literature, mothers of twins generally have higher levels of depression, exhaustion, and parenting stress than mothers of only children [29,30,31], especially in the case of MC pregnancies [4]. In addition to the psychological restructuring that any twin pregnancy entails, mothers of MC twins are at risk of miscarriage throughout the pregnancy. This clinical condition overlaps with several subfields of psychology, including parenting, psychotraumatism, and grief. Trauma is defined as the perception of the occurrence of a potentially fatal event. Not only witnessing a threatening death experience, but also indirectly learning that a close relative or friend has been exposed to trauma can lead to PTSD, according to DSM-V5. A threat to the babies’ lives during pregnancy can have the same effect on the mother. The announcement of twins in early pregnancy is often a shock that changes a parent perception. While the child had been imagined alone, the presence of two twins must now be acknowledged, along with the fears, stress, and projections that twin pregnancies can cause [32]. These psychological difficulties may increase the prevalence of major depressive disorder in twin pregnancies by up to 33.3 percent, which is almost double that of singleton pregnancies [4]. Fear of physical changes associated with this pregnancy and a negative attitude toward twins are associated with this depressive dimension [33]. After this announcement, this pregnancy is immediately classified as a ‘high-risk pregnancy’. This classification is further reinforced by the explanation of monochorionicity with all the associated risks and complications. The pregnant woman is inundated with follow-up tests that are fraught with uncertainty and doubt about the course of the pregnancy. This could be another potential risk factor for the development of a close and positive relationship with both babies as it is well documented that the presence of negative mood states, such as depression and exhaustion, in the perinatal period can affect the quality of the building of the mother–infant relationship [33,34,35,36,37,38,39].

### Objectives

The aim of the present study was to examine the quality of mothers’ behaviour during mother–infant interaction in the postpartum period by comparing mothers of twins and mothers of singletons during face-to-face interaction with their infants. Specifically, the study examined the extent to which mothers of MC twins:

- responded to their infant’s signals during the interaction;
- stimulated and engaged with their infants during the interaction;
- showed signs of depression during the interaction.

We hypothesised that mother–twin infant dyads would exhibit poorer interaction quality than mother–singleton dyads. Specifically, we hypothesised that mothers of MC twins would:

- be less sensitive and responsive to their infants’ cues during interactions with their infants [40];
- be less stimulating and less responsive during interactions with their infants [40];
- show more signs of depression during direct interactions than mothers of singletons [29,30,31].

## 2. Materials and Methods

### 2.1. Participants

Participants were recruited from a larger longitudinal case-control observational study aimed at investigating the role of mothers and fathers on neuropsychological developmental outcomes of healthy preterm infants from birth to preschool age. Between March 2013 and June 2017, parents of preterm infants were interviewed within 7–14 days after delivery in the neonatal intensive care unit of Vittore Buzzi Children’s Hospital (ASST Fatebenefratelli Sacco, Milan, Italy), while parents of term infants were interviewed within 7–14 days after delivery in the maternity ward of the same hospital. The inclusion criterion for the preterm sample was infant GA ≤ 37 weeks. Exclusion criteria for both groups were congenital anomalies, severe sensory impairment, severe brain injury or other neurological complications and parents’ lack of Italian language skills. Of the 234 infants—117 preterm (mean EG = 29.5, DS = 3.43; mean birth weight = 1254.27, DS = 470.58) and 117 term (mean EG = 38.8, DS = 1.59; mean birth weight = 3191.34, DS = 674.73)—who met the study inclusion criteria, 29 were twin pairs of MC. After recruitment, 18 twin pairs refused to participate in the study follow-up. This resulted in 11 twin pairs from MC twins participating in the second part of the original longitudinal study. Each mother of a twin was compared to a singleton mother, taking into account the gestational age at birth of their babies to eliminate the effect of prematurity and the sex of the infant.

Table 1 shows the demographic data and information about the birth and the infants’ medical data for the two groups, providing inferential statistics on differences and similarities between the groups. Maternal demographic characteristics did not differ significantly between mothers of twins and mothers of singletons, although they did not match. However, mothers of twins differed from mothers of singles when we considered mode of delivery. Specifically, all twins were delivered by caesarean section, while 40% of singletons were delivered naturally and 60% of singletons were delivered by caesarean section, as recommended for MC twins [41].

### 2.2. Procedure

All study procedures were reviewed and approved by the Ethics Committee of the Vittore Buzzi Children’s Hospital (Milan, Italy). After consenting to participate during their children’s hospitalisation, demographic and clinical information was collected by trained research psychologists from the mothers’ and children’s clinical records and from interviews with the mothers.

The three-month study visits were scheduled based on the infant’s age corrected for prematurity and at a time when the infant was most likely to be awake and alert. These study visits took place in the participants’ homes. The visit began with an initial familiarisation period for the mothers and the infants. Once the experimenter felt that the infant was comfortable, face-to-face interactions between the mother and her infant were videotaped. In the case of twins, the interactions were recorded separately for each twin.

The interactions were filmed in accordance with the procedure of the Global Rating Scales (GRS) [42,43]. The recordings were made so that a full-face image of the infants was captured, as well as a profile image of the mother. In addition, a mirror was placed next to the infant so that the mother’s entire face image could be seen in the camera image. Before the interaction began, the procedure was explained to the mother, and she was told not to use a pacifier during the interactions (unless absolutely necessary). Interactions lasted 5 min, but mothers were advised that the session could be terminated at any time if the infant became agitated or distressed or the mother became uncomfortable. Maternal behaviour during interactions was assessed and rated by two trained research psychologists using the Global Rating Scales (GRS) [42,43]. Raters were trained on to achieve >90% reliability, and any uncertainties in ratings were resolved through discussion.

### 2.3. Measures

Global Rating Scales of Mother-Infant Interaction (GRS)

The mothers’ interactive behaviours were observed, videotaped and coded using the Global Rating Scales [42,43]. This instrument has been shown to be sensitive to impaired interaction even in low-risk samples [43]. The GRS is a video-based assessment of the quality of mother–infant interaction between 2 and 4 months in the postnatal period. It has been widely used to discriminate between a variety of infant and mother populations (e.g., clinical groups with schizophrenia, social disadvantage) and has demonstrated good reliability and validity. In the present study, only the Maternal Scales were considered and assessed. As shown in Table 2, the Maternal Scale can be divided into three main dimensions: good to poor, intrusive to remote and depressive symptoms. All dimensions were composed of different subscales which were rated on a five-point scale, where 5 corresponded to optimal maternal interaction behaviour and 1 to poor maternal interaction behaviour. Inter-rater reliability was measured with Cohen’s kappa using SPSS. All Cohen’s kappa (κ) values for reliability are presented in Table 2. According to Landis and Koch’s [44] characterization of different κ-value ranges, κ > 0.75 can represent excellent, 0.40 < κ < 0.75 can represent moderate to good agreement and κ < 0.40 can represent poor agreement beyond chance. Notably, the κ coefficients for each GRS sub-scale were 0.87 or higher.

### 2.4. Analysis

Data analysis was performed in three different steps. Due to the limited sample size, non-parametric tests were used. First, the differences in the mother’s behaviour during the interaction with each twin were analysed using the Wilcoxon signed-rank test [45]. To assign the twins to two different groups, the variable birth weight was considered. Research suggests that mothers of twins prefer the sicker or the smaller twin [12,46,47,48,49], while other studies observed that mothers preferred the healthier twin [49]. In several studies, poor health at birth has been operationalized as low birth weight [50]. For this reason, the babies with higher birth weight were considered as Twin A, and the babies with lower birth weight were considered as twin B. Second, since no differences were found between the mother’s behaviour during the interactions with each twin considering all GRS subscales (see Results section), the arithmetic mean was used to create a synthetic index of the mother’s GRS [42,43] scores for each subscale [51,52]. Third, to investigate possible differences between mothers of twins and mothers of singletons, the Mann–Whitney test [53] was administered.

## 3. Results

### 3.1. Comparisons between Mother’s Behaviour during the Interaction with Each Twin

Differences in mother’s behaviour during the interaction with each twin were analysed with the Wilcoxon signed-rank test [45]. Table 3 shows GRS [42,43] scores obtained in each subscale by mothers during the interaction with Twin A (the babies with higher birth weight) and during the interaction with Twin B (the babies with lower birth weight). In the same table, non-parametric statistics about differences and similarities were reported.

No differences were found between mothers’ scores in any GRS subscale, indicating that mothers did not interact differently with their twins. This result allowed us to create a synthetic index of the mother’s scores for each subscale of the GRS using the arithmetic mean [51,52], obtaining a single score for each subscale of the GRS.

### 3.2. Comparisons between Mothers of MC Twins and Mothers of Singletons

Differences between mothers of MC twins and mothers of singletons were analysed with the Mann–Whitney test [53]. Table 4 shows GRS [42,43] scores obtained in each subscale by mothers of twins (expressed by synthetic indexes obtained from the arithmetic mean of the mother’s scores for each subscale of the GRS) and by mothers of singletons during the interaction with their babies. The same table reports non-parametric statistics about differences and similarities.

Some significant differences were found between mothers of MC twins and mothers of singletons in term of maternal behaviour during the interactions with their children. The quality of maternal sensitive behaviour during the interactions were poorer in mothers of twins. In particular, mothers of twins were less warm, less positive and less responsive during the interactions than mothers of singletons. Mothers of twins were also more remote during the interactions with their babies. Finally, mothers of twins showed higher levels of signs of depression than mothers of singletons during the interactions with their infants. In particular, it was observed that mothers of twins interacted with lower levels of energy and were less absorbed in the infants.

## 4. Discussion

Theories of neuropsychological and social–emotional growth have been proposed as the basis for children’s adaptation to the mother–child relationship [54,55]. The mother’s empathic style during the infant’s first year of life is considered a secure foundation for child development [56]. Several factors can directly influence maternal sensitivity and parenting practises, which in turn can affect the quality of mother–child interactions, such as: sleep deprivation, higher levels of depression [57], parenting stress [30,31] and difficulties in developing appropriate attachment with both children simultaneously [58,59].

All of these factors may contribute to a deterioration of the mother–child relationship; however, few studies have examined the extent to which twin status affects mother–infant interactions. Based on this gap, the overall aim of the present study was to examine the quality of mothers’ behaviour in the postpartum period by comparing mothers of MC twins and mothers of singletons during face-to-face interactions with their infants. It was hypothesised that mothers of twin infants would exhibit poorer maternal behaviours than mothers of singleton infants during interactions, and some evidence was found to support this hypothesis.

The first aim of the present study was to assess the quality of maternal behaviours related to sensitivity and responsiveness during interactions with their MC twins at 3 months of age. The results showed that mothers of MC twins responded to their infants’ cues in ways that were less appropriate to the infants’ behaviour than mothers of singleton infants. Specifically, it was observed that mothers of twins were less warm and positive than mothers of singletons and showed less love and affection toward their infants when they interacted face-to-face. Although both mothers of twins and mothers of singletons displayed a warm and affectionate tone of voice, twin mothers appeared to make fewer positive comments toward the infant and were less likely to hold and touch the infant compared to mothers of singletons. Additionally, although both mothers responded well to their children, twin mothers seemed to miss the child’s expression and behaviour more and imitate it less than mothers of only children. It was also found that mothers of MC twins were more demanding during interactions than mothers of only children, and in more cases wondered if the children were behaving in a way that was consistent with their idea of a “good child”. These findings are consistent with previous studies in which it was observed that mothers of twins were less sensitive to their babies’ cues during interactions [40]. Interestingly, however, our results show that maternal behaviour remained quite stable during interactions in twins, although different from that of mothers of singletons. As mentioned earlier, studies of prenatal attachment in twin pregnancies have found that zygosity is an important factor in the establishment of mother–infant attachment [10,11]. Women expecting identical twins tended to imagine the twins as a pair with similar characteristics [12]. Thus, we propose that mothers of MC twins were more likely to structure the mother–child relationship around the “twin pair”, without focusing on the signals and needs of the individual twins.

This study also examined the extent to which mothers of MC twins stimulated and engaged with their infants during face-to-face interactions. Mothers of MC twins were found to withdraw from their infants more than mothers of singletons. Compared to mothers of only children, mothers of twins actually seemed to be less aware of their children during the interaction. Specifically, it appears that on a few occasions, they appeared more distant from their infant, observing rather than interacting with the baby. This means that mothers of twins were more likely to act as “*observers*” when interacting with their babies than mothers of singletons. In other research, it has been observed that mothers of twins showed less initiative toward their infant during dyadic interactions than mothers of only children [39], made less physical and psychological contact and spoke less to their twins during interactions [60]. Our results show that, compared to mothers of only children, mothers of twins appeared less animated and excited during interactions with infants and more often missed opportunities to respond promptly to the infant’s needs. In addition, they did not appear to be fully engaged with the infant during the interaction. On many occasions, mothers of twins talked about things other than the immediate interaction, suggesting that the mother’s thoughts were not fully focused on the interaction with the infant. This finding is consistent with the findings of Thorpe et al. [36]. They observed that mothers of twins were less engaged and involved during interactions with their babies. Other observational studies indicated that twin infants were more likely to be interrupted than singletons and that parents of twins were less likely to focus on their infants during interactions [37,38]. This could be due to the fact that mothers of twins interact with two infants at the same time in everyday parenting and divide their attention between two infants.

Finally, the third aim of the present study was to investigate whether mothers of MC twins show more signs of depression during face-to-face interactions. It is well documented that depression in the postpartum period can interfere with the establishment of a positive mother-infant relationship [61]. Studies with preterm infants have shown that negative emotions of parents can negatively influence maternal behaviour during mother–infant interactions [19,20,21]. As noted earlier in this study, mothers of twins have historically experienced higher levels of depression, exhaustion and parenting stress than mothers of only children [29,30,31]. In particular, several studies suggest that mothers of twins generally have higher levels of depression than mothers of singletons [29,30,31], especially in the case of MC pregnancies [4]. The results of the present work suggest that mothers of MC twins showed more signs of depression during the interaction than mothers of singletons, and that mothers of twins seemed to interact with the infants with less effort and vitality than mothers of singletons. This can be explained by the fact that signs of depression are also associated with fatigue and lower energy [62]. Several studies have shown that mothers of twins generally have higher levels of fatigue, a factor that has been identified as a predictor of postpartum depression [63,64]. The results also suggest that mothers of twins, as previous research suggests [65], are less focused on the child during interactions with their twins. The difficulties associated with caring for two infants at the same time may lead mothers of twins to focus on managing more focused interactions and, consequently, to focus more on the mother. It is important to note that mother-centred behaviour during interactions may be a risk factor for infant development. Indeed, it has been demonstrated that this type of behaviour during mother–child interaction can have a negative effect on children’s language acquisition [66].

## 5. Implications

The combination of medical and psychological vulnerability associated with the birth of twins should be viewed with concern. As mentioned earlier, the birth of twins poses significant risks. Both instrumental interventions during labour and preterm birth are more common in women with MC twin births, and this may be associated with different psychological costs [67,68]. Several studies have shown that women who underwent both emergency and elective caesarean section had higher levels of anxiety and post-traumatic stress symptoms [69,70,71].

In addition, mothers of twins have to interact with two infants at the same time, which may be an additional risk factor for the infant. Twin status has been shown to be associated with language delay and impairment. Thorpe et al. [72] pointed out that the differences in language development between twins and singletons, in the absence of disability, are most likely explained by twinship in early mother–infant interaction. Based on these considerations, our findings offer an important contribution to the assessment of the impact of twin status on early mother–infant relationships. Findings from the existing literature combined with the results of the present study suggest that it is worthwhile to consider interventions with mothers and their infants after a twin birth. The poorer quality of maternal behaviour during interactions suggests that mothers of twins may benefit from interventions to promote effective interactions with their infants [25]. The World Health Organisation (WHO) [73] recommends that health professionals support parents in building healthy early relationships. Brief standardised parenting programmes that focus on sensitivity and responsiveness to the child have a positive impact on maternal psychosocial functioning and mother–child interactions [74]. In particular, methods using video feedback during home visits to a selected at-risk population of families have shown positive short-term effects on infants’ responsiveness to their mothers, as well as on parental stress and confidence in the parenting role [75,76,77].

## 6. Limitations and Future Directions

Like previous studies of mother–infant interactions after twin births [39,40] our study had a small sample size. Therefore, the study did not have the power to detect small differences, which limits generalisation from the results and the strength of conclusions. However, the study was strengthened by the fact that the groups were well matched for child and maternal characteristics. Mothers of MC but not DC twins participated in this study. It would be important to increase the number of participants and include mothers of DC twins to investigate the role of zygosity in establishing the relationship between the mother and her twins [10,11].

Furthermore, the cross-sectional data in this report cannot show how consistent these differences are between mothers of twins and mothers of singletons over time. For this reason, a longitudinal design would provide a clearer view of this aspect. A longitudinal design would also allow us to measure the impact of mothers’ behaviours during interactions with their twins on cognitive, language and emotional outcomes later in children’s development.

In this study, a standardised, reliable, and well-validated procedure was used to assess maternal behaviour during interactions. However, the use of an observational method does not allow controlling for other relevant environmental variables, such as pregnancy and hospital experience, perceived social support from partner and family, parental stress, negative parental mood and postpartum depression. It will be important to include a new type of measurement tools in the research design, such as self-report, to measure all these relevant variables.

Finally, the present study did not examine maternal behaviour during triadic interactions with either twin. Examining these triadic interactions and how mothers of twins divide their attention offer further interesting research opportunities and clinical perspective.

## Figures and Tables

**Table 1:** Maternal demographic characteristics and information about delivery.

			Twin (*N* = 11)	Singleton (*N* = 11)	Differences
Maternal age (years)	M (SD) range		38.5 (3.89) 33.00–45.00	35.7 (6.32) 28.00–46.00	*t* = 1.194, *p* = 0.247
Maternal parity	%	Primiparous	72.7%	81.8%	*χ*^2^ = 0.259, *p* = 0.611
		Pluriparous	27.3%	18.8%	
Maternal education	%	Secondary school	20%	20%	*χ*^2^ = 0.000, *p* = 1.000
		High school	20%	20%	
		Master’s	60%	60%	
Type of delivery	%	Natural	0%	40%	*χ*^2^ = 5.029, *p* = 0.025
		Caesarean	100%	60%	
GA (weeks)	M (SD) Range		34.0 (3.41) 27.00–38.20	34.0 (3.95) 27.00–38.00	*t* = 0.225, *p* = 0.824
Birth Weight (g)	M (SD) range		2003.7 (711.8) 789.00–2700.00	2045.4 (1105.1) 798.00–3650.00	*t* = −0.132, *p* = 0.896
Apgar scores 5′	M (SD) range		8.6 (1.01) 6–10	7.8 (0.50) 7–8	*t* = 1.686, *p* = 0.111

**Table 2:** Global Rating Scale maternal dimensions.

Scale	Subscales		Cohen’s Kappa
	5—Optimal	1—Poor	
Good–Poor	Warm/Positive	Cold/Hostile	0.90
	Accepting	Rejecting	0.91
	Responsive	Unresponsive	0.90
	Non-Demanding	Demanding	0.95
	Sensitive	Insensitive	0.92
Intrusive to Remote	No Intrusive Behaviour	Intrusive Behaviour	0.97
	No Intrusive Speech	Intrusive Speech	0.98
	Non Remote	Remote	0.90
	Non Silent	Silent	0.97
Signs of Depression	Happy	Sad	0.93
	Much energy	Low energy	0.95
	Absorbed in Infant	Self-Absorbed	0.97
	Relaxed	Tense	0.98

**Table 3:** Comparison between mother’s GRS scores with each twin.

	Twin A (*N* = 11)	Twin B (*N* = 11)	Test Statistics	Sig.	Eta Squared
	M (SD)	M (SD)	Z		
Good–Poor	4.00 (0.75)	4.04 (0.51)	0.000	1.000	0.13
Warm/Positive to Cold Hostile	3.91 (0.83)	3.82 (0.87)	−0.447	0.655	0.57
Accepting to Rejecting	3.91 (0.83)	4.36 (0.67)	−1.890	0.059	0.13
Responsive to Unresponsive	4.09 (1.14)	4.00 (0.78)	−0.447	0.655	0.11
Non-demanding to Demanding	4.09 (0.94)	4.00 (0.63)	−0.378	0.705	0.00
Sensitive to Insensitive	4.00 (1.00)	4.00 (0.63)	0.000	1.000	0.50
Intrusive–Remote	−0.05 (0.49)	−0.22 (0.28)	−1.715	0.086	0.00
Non-intrusive to Intrusive behaviour	4.09 (1.04)	3.73 (1.10)	−1.633	0.102	0.34
Non-intrusive to Intrusive speech	4.36 (0.67)	4.36 (0.92)	0.000	1.000	0.11
Non-remote to Remote	4.09 (0.83)	4.36 (0.81)	−1.134	0.257	0.13
Non-silent to Silent	4.55 (0.69)	4.64 (0.67)	−0.378	0.705	0.25
Signs of Depression	3.89 (0.57)	4.07 (0.56)	−1.372	0.170	0.52
Happy to Sad	4.09 (0.70)	4.18 (0.60)	−0.447	0.655	0.30
Much Energy to Low Energy	3.73 (0.91)	3.91 (0.83)	−0.816	0.441	0.13
Absorbed in Infant to Self-absorbed	3.73 (0.79)	4.00 (0.76)	−1.732	0.083	0.57
Relaxed to Tense	4.00 (0.78)	4.18 (0.75)	−1.000	0.317	0.13

Note. GRS scores are rated on a 5-point Likert scale (5 = optimal–1 = poor).

**Table 4:** Comparisons between GRS scores obtained by mothers of MC twins and by mothers of singletons.

	Mother of MC Twin (*N* = 11)	Mother of Singleton (*N* = 11)	U of Mann–Whitney	Sig.	Eta Squared
	M (SD)	M (SD)			
Good–Poor	4.02 (0.59)	4.60 (0.37)	23.00	0.012	0.35
Warm/Positive to Cold Hostile	3.86 (0.77)	4.73 (0.47)	21.50	0.004	0.09
Accepting to Rejecting	4.14 (0.67)	4.55 (0.52)	41.00	0.232	0.20
Responsive to Unresponsive	4.05 (0.91)	4.73 (0.47)	31.50	0.040	0.11
Non-demanding to Demanding	4.05 (0.69)	4.45 (0.52)	38.00	0.111	0.17
Sensitive to Insensitive	4.00 (0.71)	4.55 (0.52)	33.00	0.079	0.30
Intrusive–Remote	−0.13 (0.36)	−0.27 (0.33)	47.50	0.397	0.02
Non-intrusive to Intrusive behaviour	3.91 (1.02)	4.09 (0.70)	57.50	0.853	0.02
Non-intrusive to Intrusive speech	4.36 (0.74)	4.55 (0.52)	52.50	0.634	0.26
Non-remote to Remote	4.23 (0.72)	4.82 (0.40)	28.50	0.025	0.15
Non-silent to Silent	4.59 (0.54)	4.91 (0.30)	39.00	0.126	0.04
Signs of Depression	3.98 (0.52)	4.52 (0.44)	25.00	0.016	0.05
Happy to Sad	4.14 (0.55)	4.36 (0.81)	46.00	0.358	0.38
Much Energy to Low Energy	3.81 (0.78)	4.73 (0.47)	20.00	0.004	0.21
Absorbed in Infant to Self-absorbed	4.09 (0.70)	4.55 (0.52)	30.50	0.044	0.08
Relaxed to Tense	3.98 (0.52)	4.52 (0.44)	42.00	0.209	0.27

Note. GRS scores are rated on a 5-point Likert scale (5 = optimal–1 = poor).

## Data Availability

The dataset is not publicly available due the hospital privacy policy.
